# A Simplified and Versatile System for the Simultaneous Expression of Multiple siRNAs in Mammalian Cells Using Gibson DNA Assembly

**DOI:** 10.1371/journal.pone.0113064

**Published:** 2014-11-14

**Authors:** Fang Deng, Xiang Chen, Zhan Liao, Zhengjian Yan, Zhongliang Wang, Youlin Deng, Qian Zhang, Zhonglin Zhang, Jixing Ye, Min Qiao, Ruifang Li, Sahitya Denduluri, Jing Wang, Qiang Wei, Melissa Li, Nisha Geng, Lianggong Zhao, Guolin Zhou, Penghui Zhang, Hue H. Luu, Rex C. Haydon, Russell R. Reid, Tian Yang, Tong-Chuan He

**Affiliations:** 1 Department of Cell Biology, Third Military Medical University, Chongqing, 400038, China; 2 Molecular Oncology Laboratory, Department of Orthopaedic Surgery and Rehabilitation Medicine, The University of Chicago Medical Center, Chicago, IL, 60637, United States of America; 3 Department of Orthopaedic Surgery, the Affiliated Xiang-Ya Hospital of Central South University, Changsha, 410008, China; 4 Ministry of Education Key Laboratory of Diagnostic Medicine, and the Affiliated Hospitals of Chongqing Medical University, Chongqing, 400016, China; 5 Department of Surgery, the Affiliated Zhongnan Hospital of Wuhan University, Wuhan, 430071, China; 6 School of Bioengineering, Chongqing University, Chongqing, 400044, China; 7 Department of Orthopaedic Surgery, the Second Affiliated Hospital of Lanzhou University, Lanzhou, Gansu, 730000, China; 8 The Laboratory of Craniofacial Biology, Department of Surgery, The University of Chicago Medical Center, Chicago, IL, 60637, United States of America; Rush University Medical Center, United States of America

## Abstract

RNA interference (RNAi) denotes sequence-specific mRNA degradation induced by short interfering double-stranded RNA (siRNA) and has become a revolutionary tool for functional annotation of mammalian genes, as well as for development of novel therapeutics. The practical applications of RNAi are usually achieved by expressing short hairpin RNAs (shRNAs) or siRNAs in cells. However, a major technical challenge is to simultaneously express multiple siRNAs to silence one or more genes. We previously developed pSOS system, in which siRNA duplexes are made from oligo templates driven by opposing U6 and H1 promoters. While effective, it is not equipped to express multiple siRNAs in a single vector. Gibson DNA Assembly (GDA) is an *in vitro* recombination system that has the capacity to assemble multiple overlapping DNA molecules in a single isothermal step. Here, we developed a GDA-based pSOK assembly system for constructing single vectors that express multiple siRNA sites. The assembly fragments were generated by PCR amplifications from the U6-H1 template vector pB2B. GDA assembly specificity was conferred by the overlapping unique siRNA sequences of insert fragments. To prove the technical feasibility, we constructed pSOK vectors that contain four siRNA sites and three siRNA sites targeting human and mouse β-catenin, respectively. The assembly reactions were efficient, and candidate clones were readily identified by PCR screening. Multiple β-catenin siRNAs effectively silenced endogenous β-catenin expression, inhibited Wnt3A-induced β-catenin/Tcf4 reporter activity and expression of Wnt/β-catenin downstream genes. Silencing β-catenin in mesenchymal stem cells inhibited Wnt3A-induced early osteogenic differentiation and significantly diminished synergistic osteogenic activity between BMP9 and Wnt3A *in vitro* and *in vivo*. These findings demonstrate that the GDA-based pSOK system has been proven simplistic, effective and versatile for simultaneous expression of multiple siRNAs. Thus, the reported pSOK system should be a valuable tool for gene function studies and development of novel therapeutics.

## Introduction

RNA interference (RNAi) was first discovered in *C. elegans* as a protecting mechanism against invasion by foreign genes and has subsequently been demonstrated in diverse eukaryotes, such as insects, plants, fungi and vertebrates [1]–[7]. RNAi is a cellular process of sequence-specific, post-transcriptional gene silencing initiated by double-stranded RNAs (dsRNA) homologous to the gene being suppressed. The dsRNAs are processed by Dicer to generate duplexes of approximately 21nt, so-called short interfering RNAs (siRNAs), which cause sequence-specific mRNA degradation. Dicer-produced siRNA duplexes comprise two 21 nucleotide strands, each bearing a 5′ phosphate and 3′ hydroxyl group, paired in a way that leaves two-nucleotide overhangs at the 3′ ends. Target regulation by siRNAs is mediated by the RNA-induced silencing complex (RISC). Since its discovery, RNAi has become a valuable and powerful tool to analyze loss-of-function phenotypes *in vitro* and *in vivo* [2]–[7]. Given its gene-specific targeting nature, RNAi also offers unprecedented opportunities for developing novel and effective therapeutics for human diseases [8]–[13].

The practical applications of siRNA duplexes to interfere with the expression of a given gene require target accessibility and effective delivery of siRNAs into target cells and for certain applications long-term siRNA expression [8]–[14]. While RNAi can be achieved by delivering synthetic short double-stranded RNA duplexes into cells, a more commonly-used approach is to express short hairpin RNAs (shRNAs) or siRNAs in cells [11], [12], [14]. In this case, the endogenous expression of siRNAs is achieved by using various Pol III promoter expression cassettes that allow transcription of functional siRNAs or their precursors [14]. However, one of the formidable technical challenges is to effectively construct these RNAi expression vectors, especially when gene silencing necessitates the use of multiple siRNA target sites for a gene of interest. We previously developed the pSOS system, in which the siRNA duplexes are made from an oligo template driven by opposing U6 and H1 promoters [15]. While effective, it usually requires to make multiple vectors and multiple-round infections to achieve effective knockdown when multiple siRNA sites are used. On the other hand, there are clear needs to simultaneously deliver multiple siRNAs that target more than one genes.

Gibson DNA Assembly (GDA), so named after the developer of the method [16], is one of commonly-used synthetic biology techniques that offer restriction enzyme-free, scarless, largely sequence-independent, and multi-fragment DNA assembly [17], [18]. GDA is an *in vitro* recombination system that has the capacity to assemble and repair multiple overlapping DNA molecules in a single isothermal step [16], [17]. The optimized GDA contains three essential components: an exonuclease (e.g., 5′-T5 exonuclease) that removes nucleotides from the ends of double-stranded (ds) DNA molecules so exposing complementary single-stranded (ss) DNA overhangs that are specifically annealed; a DNA polymerase (e.g., Phusion DNA polymerase) that fills in the ssDNA gaps of the joined molecules; and a DNA ligase (e.g., Taq ligase) that covalently seals the nicks [17]. Thus, this assembly method can be used to seamlessly construct synthetic and natural genes, genetic pathways, and entire genomes as useful molecular engineering tools [16]–[18].

Here, we sought to use the GDA technique to establish a simplified one-step assembly system for constructing a single vector that expresses multiple siRNA target sites. To achieve this, we have engineered the GDA destination retroviral vector pSOK, based on our previously reported pSOS vector [15], which can be linearized with SwaI for assembly reactions. The assembly fragments containing multiple siRNA sites are generated by PCR amplifications using the back-to-back U6-H1 promoter vector pB2B as a template. The first fragment overlaps with the 3′-end of U6 promoter while the last fragment overlaps with the 3′-end of H1 promoter. The ends of the middle fragments overlap the specific siRNA target sequences, which confers assembly specificity. After the GDA reactions, single vectors expressing multiple siRNA target sites are generated. To prove the feasibility of this pSOK system, we have developed the vectors that contain four siRNA sites and three siRNA sites that target human and mouse β-catenin, respectively. We demonstrate that the assembly reactions are efficient, and that candidate clones are readily identified by PCR screening, although vectors containing three siRNAs are seemingly more favorably assembled under our assembly condition. Functional analyses demonstrate that the multiple β-catenin siRNA constructs can effectively silence endogenous β-catenin expression, inhibit Wnt3A-induced β-catenin/Tcf4 reporter activity and the expression of Wnt/β-catenin downstream target genes. In mesenchymal stem cells, silencing β-catenin inhibits Wnt3A-induced early osteogenic differentiation and significantly diminishes the synergistic osteogenic activity between BMP9 and Wnt3A both *in vitro* and *in vivo*. Taken together, our results have demonstrated that the GDA-based pSOK system is proven simplistic, effective and versatile for simultaneous expression of multiple siRNA target sites. Thus, the pSOK system should be a valuable tool for gene function studies and the development of therapeutics.

## Materials and Methods

### Cell culture and chemicals

HEK-293 and human colon cancer SW480 lines were purchased from ATCC (Manassas, VA) and maintained in complete Dulbecco's Modified Eagle's Medium (DMEM) containing 10% fetal bovine serum (FBS, Invitrogen, Carlsbad, CA), 100 units of penicillin and 100 µg of streptomycin at 37°C in 5% CO_2_ [19]–[24]. The reversibly immortalized mouse embryonic fibroblasts (iMEFs) were previously characterized [25], [26]. The recently engineered 293pTP line was used for adenovirus amplification [27]. Both 293pTP and iMEFs were maintained in complete DMEM. Unless indicated otherwise, all chemicals were purchased from Sigma-Aldrich (St. Louis, MO) or Fisher Scientific (Pittsburgh, PA).

### Construction of the retroviral vector pSOK and PCR template vector pB2B for Gibson DNA Assembly reactions

**Figure 1:**
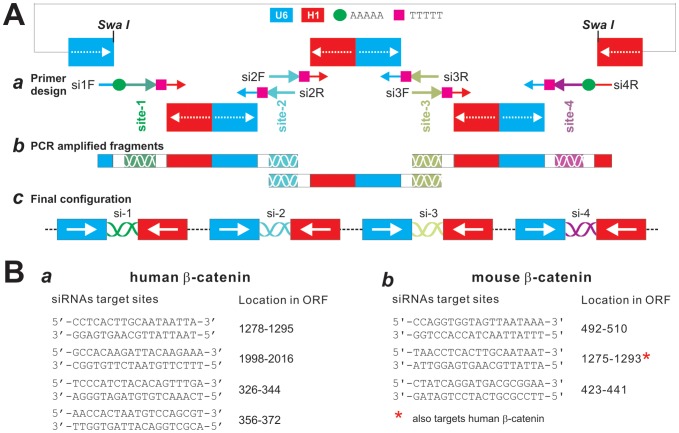
Schematic depiction of the one-step system pSOK for expressing multiple siRNAs. (**A**) Schematic representation of a tandem siRNA targeting configuration (4 sites listed as an example). The pSOK vector was constructed based on the previously reported pSOS vector, which contains opposing U6 and H1 promoters to drive siRNA duplex expression (**Figure S1A**) [15]. The linker sites of the pSOS vector were modified and a SwaI site was created for linearizing the vector for Gibson Assembly (***a***). The primers were designed according to the guidelines outlined in **Figure S2A**. Using the pB2B as a template vector (**Figure S1B**), the back-to-back U6-H1 promoter fragments with different siRNA target sites were generated. The first fragment overlaps with the 3′-end of the U6 promoter while the last fragment overlaps with the 3′-end of the H1 promoter (***b***). The ends of the middle fragments overlaps the specific siRNA target sequences (***b***). After the Gibson Assembly reaction, a single vector expressing 4 siRNA target sites is constructed (***c***). It is noteworthy that the siRNA sites may target the same or different genes. (**B**) The targeting sequences and locations of the designed siRNA sites on human (***a***) and mouse (***b***) β-catenin open reading frame (ORF). All of these sites have been validated in previous studies [15], [40]. Note that one of the mouse siRNAs also targets human β-catenin coding sequence.

As illustrated in **Figure 1A** and **Figure S1A**, the MSCV retroviral vector pSOK was constructed on the base of our previously reported pSOS vector [15], which contains the opposing U6 and H1 promoters to drive siRNA duplex expression. The linker sites of the pSOS vector were modified and a SwaI site was engineered for linearizing the vector for Gibson Assembly (**Figure 1A** **, panel** ***a***). This vector also confers Blasticidin S resistance for generating stable mammalian cell lines. The pB2B vector was constructed on the base of our previously reported pMOLuc vector [28]. Briefly, the high-fidelity PCR amplified U6 and H1 promoter fragments were subcloned into the EcoRI/HindIII sites of pMOLuc in a back-to-back orientation, and ligated at MluI site (**Figure S1B**). Both U6 and H1 promoters contain a string of “AAAAA” immediately preceding their transcription start sites, which serves as transcription termination signal for the reverse strand. The full-length vector sequences and maps are available at: http://www.boneandcancer.org/MOLab%20Vectors%20after%20Nov%201%202005/pSOK.pdf and http://www.boneandcancer.org/MOLab%20Vectors%20after%20Nov%201%202005/pBOK%20vector%20map%20and%20sequence%202013-12-02.pdf.

### Gibson DNA Assembly (GDA) reactions for generating pSOK vectors expressing siRNA sites targeting human and mouse β-catenin and the generation of stable cell lines

The GDA reactions were carried out by using the Gibson Assembly Master Mix from New England Biolabs (Ipswich, MA) following the manufacturer's instructions. The overlapping inserts were prepared by PCR amplifications using the Phusion High-Fidelity PCR kit (New England Biolabs). Each assembly reaction contained approximately 100 ng of each insert and 50 ng of the SwaI-linearized pSOK vector and incubated at 50°C for 45 min. After the assembly reactions, the reaction mix was briefly digested with SwaI and transformed into electro-competent DH10B cells. Colony PCR screening was carried out using a forward and reverse primer pair of the two neighboring siRNA sites. Positive clones were sequencing verified. Regardless the compositions of the obtained clones, vectors containing one, two, three or four siRNA sites targeting human β-catenin were designated as pSOK-siBC1, pSOK-siBC2, pSOK-siBC3, and pSOK-siBC4, respectively. For the mouse β-catenin siRNAs, we only chose the vector that contains all three siRNA sites, namely pSOK-simBC3 for this study. A control vector containing three scrambled sites that do not target any human and mouse genes (5′-GCAAAGACGCAATAATACA-3′; 5′-GCACAAAGAACGACTATAA-3′; 5′-GAAACACGATTAACAGACA-3′) was also constructed, designated as pSOK-siControl.

The stable knockdown lines were generated using a retrovirus system as previously reported [15], [29]–[31]. Briefly, the siRNA-containing pSOK vectors were co-transfected with the retrovirus packaging plasmid pCL-Ampho into HEK-293 cells. The packaged retrovirus supernatants were used to infect 293, SW480 (for siBC vectors) and iMEFs (for simBC3 vector). The infected cells were selected in Blasticidin S (4 μg/ml) for 5–7 days. The stable pools of cells were kept in LN2 for long-term storage. The resultant stable lines were designated such as 293-siBC4, 293-siControl, SW480-siBC4, SW480-siControl, iMEF-simBC3, and iMEF-siControl, to name a few.

### Generation and amplification of recombinant adenoviruses expressing BMP9, Wnt3A, and GFP

Recombinant adenoviruses were generated using the AdEasy technology as described [30], [32]–[34]. The coding regions of human BMP9 and mouse Wnt3A were PCR amplified and cloned into an adenoviral shuttle vector, and subsequently used to generate and amplify recombinant adenoviruses in HEK-293 or 293pTP cells [27]. The resulting adenoviruses were designated as AdBMP9 and AdWnt3A, both of which also express GFP [35]–[38]. Analogous adenovirus expressing only GFP (AdGFP) was used as controls [39]–[42]. For all adenoviral infections, polybrene (4–8 μg/ml) was added to enhance infection efficiency as previously reported [23].

### Cell transfection and firefly luciferase reporter assay

Subconfluent cells were transfected with the Tcf/Lef reporter pTOP-Luc using Lipofectamine Reagent (Invitrogen) by following the manufacturer's instructions. For 293 and iMEF cells, the cells were co-transfected with pCMV-Wnt3A. At 48 h post transfection, cells were lysed for luciferase assays using Luciferase Assay System (Promega, Madison, WI) by following the manufacturer's instructions. Easy conditions were done in triplicate.

### RNA isolation and quantitative real-time PCR (qPCR)

Total RNA was isolated by using TRIZOL Reagents (Invitrogen) and used to generate cDNA templates by reverse transcription reactions with hexamer and M-MuLV reverse transcriptase (New England Biolabs, Ipswich, MA). The cDNA products were used as PCR templates. The sqPCR were carried out as described [43]–[47]. PCR primers (**Table S1**) were designed by using the Primer3 program and used to amplify the genes of interest (approximately 150–250 bp). For qPCR analysis, SYBR Green-based qPCR analysis was carried out by using the thermocycler Opticon II DNA Engine (Bio-Rad, CA) with a standard pUC19 plasmid as described elsewhere [21], [48]–[50]. The qPCR reactions were done in triplicate. The sqPCR was also carried out as described [15], [24], [25], [27], [29], [39], [47], [51], [52]. Briefly, sqPCR reactions were carried out by using a touchdown protocol: 94°C×20″, 68°C×30″, 70°C×20″ for 12 cycles, with 1°C decrease per cycle, followed by 25–30 cycles at 94°C×20″, 56°C×30″, 70°C×20″. PCR products were resolved on 1.5% agarose gels. All samples were normalized by the expression level of GAPDH.

### Immunofluorescence staining

Immunofluorescence staining was performed as described [30], [40], [43], [50], [53], [54]. Briefly, cells were infected with AdWnt3A or AdGFP for 48 h, fixed with methanol, permeabilized with 1% NP-40, and blocked with 10% BSA, followed by incubating with β-catenin antibody (Santa Cruz Biotechnology). After being washed, cells were incubated with Texas Red-labeled secondary antibody (Santa Cruz Biotechnology). Stains were examined under a fluorescence microscope. Stains without primary antibodies, or with control IgG, were used as negative controls.

### Qualitative and quantitative assays of alkaline phosphatase (ALP) activity

ALP activity was assessed quantitatively with a modified assay using the Great Escape SEAP Chemiluminescence assay kit (BD Clontech, Mountain View, CA) and qualitatively with histochemical staining assay (using a mixture of 0.1 mg/ml napthol AS-MX phosphate and 0.6 mg/ml Fast Blue BB salt), as previously described [29], [30], [32], [33], [39], [40], [44], [53]. Each assay condition was performed in triplicate and the results were repeated in at least three independent experiments.

### iMEF cell implantation and ectopic bone formation

All animal studies were conducted by following the guidelines approved by the Institutional Animal Care and Use Committee (IACUC) of The University of Chicago (protocol #71108). Stem cell-mediated ectopic bone formation was performed as described [20], . Briefly, subconfluent iMEFsimBC3 and iMEF-siControl cells were infected with AdBMP9 and/or AdWnt3A, or AdGFP for 16 h, collected and resuspended in PBS for subcutaneous injection (5×10^6^/injection) into the flanks of athymic nude (nu/nu) mice (5 animals per group, 4–6 wk old, female, Harlan Laboratories, Indianapolis, IN). At 4 weeks after implantation, animals were sacrificed, and the implantation sites were retrieved for histologic evaluation and Trichrome staining as described below.

### Histological evaluation and Trichrome staining

Retrieved tissues were fixed, decalcified in 10% buffered formalin, and embedded in paraffin. Serial sections of the embedded specimens were stained with hematoxylin and eosin (H & E). Trichrome staining was carried out as previously described [20], [25], [26], [44], [47], [52], [55], [56].

### Statistical analysis

The quantitative assays were performed in triplicate and/or repeated three times. Data were expressed as mean ± SD. Statistical significances were determined by one-way analysis of variance and the student's *t* test. A value of *p*<0.05 was considered statistically significant.

## Results

### Construction of the GDA vector pSOK for expressing multiple siRNA target sites in mammalian cells

We previously developed the pSOS system, in which the siRNA duplexes are made from an oligo template driven by opposing U6 and H1 promoters [15]. While effective, it usually requires to make multiple vectors and multiple-round infections to achieve effective knockdown if multiple siRNA target sites are used. In other cases, there are clear needs to deliver multiple siRNAs that target more than one genes. Here, we sought to establish a simplified one-step approach, based on the GDA technology, which will allow us to make a single vector that express multiple siRNA target sites against one gene or multiple genes.

As depicted in **Figure 1A**, the pSOK vector was constructed based on the previously reported pSOS vector [15], which contains the opposing U6 and H1 promoters to drive siRNA duplex expression (**Figure S1A**). The linker sites of the pSOS vector were modified and a SwaI site was engineered for linearizing the vector for Gibson Assembly (**Figure 1A** **, panel** ***a***). This vector confers Blasticidin S resistance for generating stable mammalian cell lines. For examples, four siRNA target sites are exemplified to illustrate the primer design and construction process, the primers were designed according to the guidelines outlined in **Figure S2A**. Using the pB2B as a template vector (**Figures S1B, and S2B**), the back-to-back U6-H1 promoter fragments with different siRNA target sites were generated. The first fragment overlaps with the 3′-end of the U6 promoter while the last fragment overlaps with the 3′-end of the H1 promoter (**Figure 1A** **, panel** ***b***). Thus, the ends of the middle fragments overlap the specific siRNA target sequences (**Figure 1A** **, panel** ***b***). After the GDA reaction, a single vector expressing four siRNA target sites is constructed (**Figure 1A** **, panel** ***c***).

To prove the principle and feasibility of the pSOK system, we designed four siRNA sites and three siRNA sites that target human and mouse β-catenin, respectively (**Figure 1B**). The targeting sequences and locations of the designed siRNA sites on human (**Figure 1B** **, panel** ***a***) and mouse (**Figure 1B** **, panel** ***b***) β-catenin open reading frame (ORF). These siRNA sites were previously demonstrated to effectively silence β-catenin expression [15], [40]. As indicated, these siRNA sites target a broad region of β-catenin coding regions, and one of the mouse siRNAs also target human β-catenin (**Figure 1A** **, panel** ***b***).

### Construction and characterization of pSOK vectors that express multiple siRNAs targeting human β-catenin

**Figure 2:**
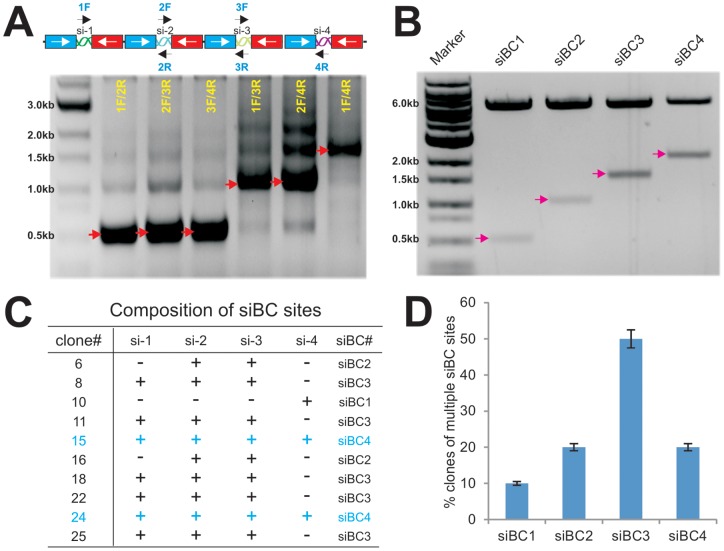
Construction and characterization of pSOK vectors that express multiple siRNAs targeting human β-catenin. (**A**) PCR screening strategy for candidate clones. A representative pSOK-siBC4 clone was used as a template and PCR amplified with different combinations of primer pairs, as depicted above the gel image. The PCR products were resolved on a 0.8% agarose gel. The arrows indicate the expected products. (**B**) Restriction digestion confirmation of the obtained clones. Representative clones containing 1 to 4 siRNA sites were digested with PmeI/HindIII to release the inserts. The digested products were resolved on a 0.8% agarose gel. The arrows indicate the expected products. (**C**) The frequency of the clones containing multiple copies of siRNA target expression units of human β-catenin. Plasmid DNA was isolated from approximately 80 individual SwaI-resistant clones and subjected to DNA sequencing. The presence of different copy numbers of siRNA sites was tabulated. (**D**) siRNA target site composition of 10 representative clones of human β-catenin. “+”, site present, “−”, site absent.

We first chose to construct the pSOK vector expressing the four siRNAs that target human β-catenin. After performing PCR amplifications of the three inserts as depicted in **Figure 1A**, we carried out the GDA reactions using the three inserts and the SwaI-linearized pSOK vector. The potential recombinants were screened by colony PCR using the forward and reverse primer pairs of the neighboring siRNA sites (**Figure 2A**). Since there were multiple repetitive U6-H1 promoter units in the construct, we found the most robust and specific amplifications were obtained when the forward and reverse primer pairs of the neighboring siRNA sites were used. To further demonstrate this phenomenon, we used a representative pSOK-siBC4 clone as the template and tested PCR amplifications with different combinations of primer pairs. We found that the primer pairs 1F/2R, 2F/3R, and 3F/4R yielded robust and relatively specific products while primer pairs covering two or more siRNA sites produced multiple bands (**Figure 2A**).

Since the overlapping sequences for the inserts are only 19 bp, it is conceivable that a high exonuclease activity in the assembly reaction may over digest the overlapping sequences and cause mis-pairing of the siRNA sites. In fact, we found clones containing one to four siRNA sites as shown by the restriction digestion to release the inserts (in roughly 500 bp U6-H1 cassettes) (**Figure 2B**). More than candidate clones were verified by DNA sequencing and blasting against the query sequence outlined in **Figure S2C**. Sequencing analysis of these clones revealed that different clones may contain different siRNA sites (**Figure 2C**). Statistically, clones containing three siRNA sites (i.e, siBC3) are the most abundant (accounted for about 50% of the clones), followed by clones containing two or four siRNA sites (i.e., siBC2 or siBC4) at about 20% each (**Figure 2D**). Surprisingly, clones containing one siRNA site only accounted for about 10% of the screened clones (**Figure 2D**). These results indicate that the assembly reactions are efficient although the assembly of three siRNA sites (e.g., two inserts) may be a more favorable event, at least under our reaction conditions. After sequencing verification, the clones containing one, two, three and four siRNA sites were pooled and designated as pSOK-siBC1, pSOK-siBC2, pSOK-siBC3 and pSOK-siBC4, respectively. An analogous vector containing three scrambled sites was also constructed as a control (e.g., pSOK-siControl). While we chose to construct multiple siRNAs to target the same gene (i.e., β-catenin), it is conceivable that one can assemble multiple siRNAs that target more than one genes.

### Functional validation of the pSOK-siBC4 vector that contains four siRNA sites targeting human β-catenin

**Figure 3:**
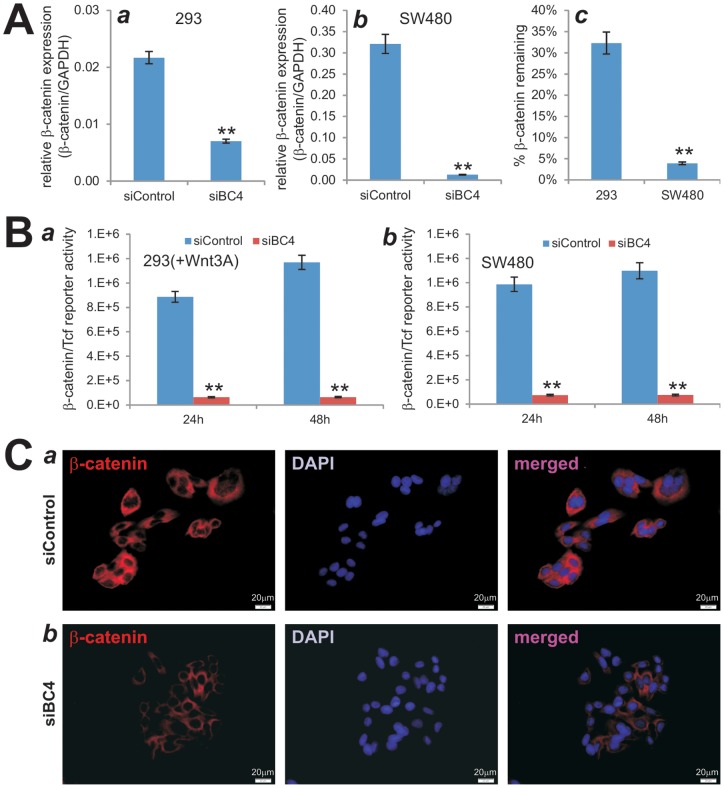
Functional validation of siRNAs targeting human β-catenin. (**A**) Efficient knockdown of endogenous β-catenin in HEK-293 and SW480 cells. Total RNA was isolated from subconfluent 293-siBC4, 293-siControl, SW480-siBC4, and SW480-siControl cells, and subjected to qPCR analysis using primers specific for human β-catenin. All samples were normalized with GAPDH. Each reaction was done in triplicate. Relative β-catenin expression was calculated by dividing β-catenin expression levels with respective GAPDH levels in 293 (***a***) and SW480 (***b***) cells. The % of remaining β-catenin expression was calculated by dividing the relative β-catenin expression in siBC4 with that of the respective siControl's (***c***). “**”, *p<0.001*. (**B**) β-Catenin/Tcf transcription activity is significantly reduced in siBC4 cells. Subconfluent 293-siBC4 and 293-siControl cells were co-transfected with TOP-Luc reporter and pCMV-Wnt3A plasmids using Lipofectamine reagent (***a***), while SW480-siBC4 and SW480-siControl cells were transfected with TOP-Luc reporter plasmid using Lipofectamine reagent (***b***). At 24 h and 48 h after transfection, the cells were lysed and subjected to firefly luciferase assay using the Luciferase Reporter Assay System (Promega). Each assay condition was done in triplicate. “**”, *p<0.001*. (**C**) siBCs can effectively block Wnt3a-induced β-catenin accumulation. Subconfluent SW480-siBC4 and SW480-siControl cells fixed and subjected to immunofluorescence staining with an anti-β-catenin antibody. The cell nuclei were counter stained with DAPI. Control IgG and minus primary antibody were used as negative controls (data not shown).

Although most of these siRNA target sites have been tested for their silencing efficiency and effect on Wnt/β-catenin signaling activity, the knockdown efficiency may be compromised due to promoter competitions in the pSOK system because multiple U6-H1 expression cassettes are engineered in a single vector. To test this possibility, we established stable lines of HEK-293 and SW480 cells expressing the siBC sites or siControl using a retroviral system. We first assessed the knockdown efficiency of endogenous β-catenin in 293 cells and SW480 cells. Using qPCR analysis, we found that the endogenous β-catenin expression in 293-siBC4 was significantly lower than that of the 293-siControl's (**Figure 3A** **, panel a**). Similarly, using the human colon cancer line SW480 we found the β-catenin expression was drastically reduced in SW480-siBC4 cells compared with that in the SW480-siControl cells (**Figure 3A** **, panel b**). Overall, the siBC4-expressing 293 and SW480 cells exhibited marked decreases in the β-catenin expression, only about 32% and 4% of the control cells' (**Figure 3A** **, panel c**). It is noteworthy that we also found that endogenous β-catenin expression was significantly reduced in the 293 and SW480 cells that express siBC1, siBC2, and siBC3 (data not shown). When the 293-siBC4 (co-transfected with Wnt3A) and SW480-siBC4 cells were transfected with the β-catenin/Tcf reporter pTOP-Luc, the reporter activities were marked reduced at the tested time points in both 293 cells (*p<0.001*) (**Figure 3B** **, panel a**) and SW480 cells (*p<0.001*) (**Figure 3B** **, panel b**). Moreover, the Wnt3A-stimulated reporter activities in 293 cells stably expressing siBC1, siBC2, and siBC3 were also remarkably inhibited (**Figure S3A**), and similar results were obtained in SW480 cells, in which the Wnt/β-catenin signaling is constitutively active (**Figure S3B**). Furthermore, we examined the β-catenin knockdown efficiency in SW480 cells by immunofluorescence staining. We found that cytoplasmic/nuclear accumulation of β-catenin in SW480-siBC4 cells was significantly diminished, compared with that in the SW480-siControl cells (**Figure 3C**). Taken the above results together, the pSOK-siBC4 vector that expresses four human β-catenin siRNA sites can effectively silence β-catenin expression in human cells.

### pSOK-simBC3 contains multiple siRNAs targeting mouse β-catenin and effectively inhibits canonical Wnt signaling activity in iMEFs

**Figure 4:**
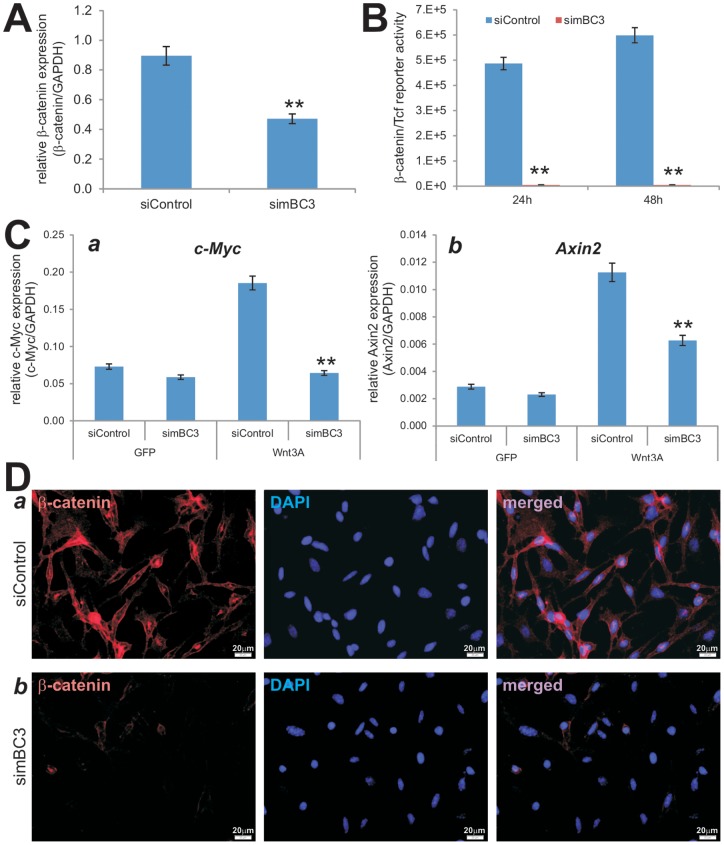
Multiple siRNAs targeting mouse β-catenin simBC3 effectively inhibit canonical Wnt signaling activity in iMEFs. (**A**) Reduced β-catenin expression in iMEF-simBC3 cells. Subconfluent iMEF-simBC3 and iMEF-siControl cells were infected with AdWnt3A or AdGFP. At 36 h after infection, total RNA was isolated and subjected to qPCR analysis using primers for mouse β-catenin and GAPDH. Relative expression was calculated by dividing the β-catenin expression levels with respective GAPDH expression. All samples were subjected to the subtraction of baseline (i.e., AdGFP infected cells) expression. Each assay was done in triplicate. “**”, *p<0.001*. (**B**) iMEF-simBC3 cells exhibit significantly lower β-catenin/Tcf reporter activity upon Wnt3A stimulation. Subconfluent iMEF-simBC3 and iMEF-siControl cells were transfected with TOP-Luc reporter plasmid and infected with AdWnt3A or AdGFP. At 24 h and 48 h post transfection/infection, cells were lysed for luciferase assays. Relative β-catenin/Tcf reporter activity was subjected to subtractions of basal activity (i.e., AdGFP groups). Easy conditions were done in triplicate. “**”, *p<0.001*. (**C**). Wnt3A-induced expression of Wnt/β-catenin target genes was significantly decreased in iMEF-simBC3 cells. Subconfluent iMEF-simBC3 and iMEF-siControl cells were infected with AdWnt3A or AdGFP for 36 h. Total RNA was isolated and subjected to reverse transcription. The resultant cDNAs were used as templates for qPCR analysis using primers specific for mouse Axin2 and c-Myc transcripts. All samples were normalized by GAPDH levels. Each assay condition was done in triplicate. “**”, *p<0.001*. (**D**) simBC3 can effectively block Wnt3a-induced β-catenin accumulation. Subconfluent iMEF-siControl (a) cells fixed and subjected to immunofluorescence staining with an anti-β-catenin antibody. The cell nuclei were counter stained with DAPI. Control IgG and minus primary antibody were used as negative controls (data not shown). Representative images are shown.

Our results in **Figure 2** indicate that three siRNA sites (two inserts) are seemingly more favorably assembled into pSOK vector. It is conceivable that in most cases three siRNA sites should be sufficiently effective in silencing a given gene. Here, we tested this possibility by constructing a vector, designated as pSOK-simBC, that expressed three siRNA sites targeting mouse β-catenin (**Figure 1B** **, panel b**). The construction and screening process were very efficient. After sequencing verification, the pSOK-simBC was packaged as retrovirus and used to generate the stable line iMEF-simBC3, along with a control line iMEF-siControl. The iMEFs were previously characterized multi-potent mesenchymal stem cells (MSCs) [20], [25]. When the iMEF stable lines were infected with AdWnt3A or AdGFP and analyzed for β-catenin expression, we found that β-catenin expression was significantly reduced in iMEF-simBC3 cells, compared with that in iMEF-siControl cells (*p<0.001*) (**Figure 4A**).

Using the β-catenin/Tcf luciferase reporter, we found that iMEF-simBC3 cells exhibited significantly lower β-catenin/Tcf reporter activity upon Wnt3A stimulation (p<0.001) (**Figure 4B**). Accordingly, when the expression of two well-characterized Wnt/β-catenin downstream target genes, Axin2 [58] and c-Myc [59], was examined, we found that Wnt3A was shown to significantly induce the expression of Axin2 and c-Myc in iMEF-siControl cells; but silencing β-catenin in iMEFs significantly diminished Wnt3A-induced expression of c-Myc (**Figure 4C** **, panel a**) and Axin2 (**Figure 4C** **, panel b**). Furthermore, immunofluorescence staining indicate that Wnt3A-induced cytoplasmic/nuclear accumulation of β-catenin protein was effectively reduced in iMEF-simBC3 cells, compared with that in iMEF-siControl cells (**Figure 4D**). Therefore, the above data demonstrate that the three-siRNA site-containing pSOK-simBC3 can effectively blunt the functional activities of Wnt3A/β-catenin in iMEF cells.

### Silencing β-catenin diminishes the synergistic osteogenic activity between BMP9 and Wnt3A in iMEF cells

**Figure 5:**
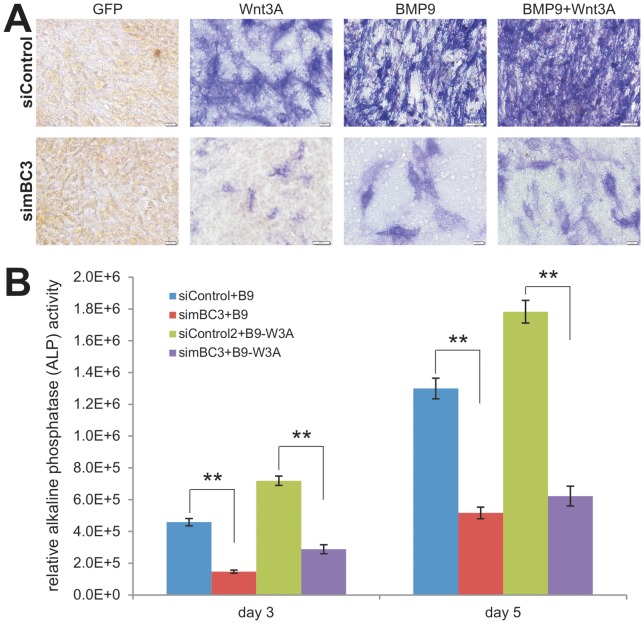
Silencing β-catenin diminishes the synergistic osteogenic activity between BMP9 and Wnt3A in iMEF cells. (**A**) Wnt3A and/or BMP9-induced early osteogenic marker alkaline phosphatase (ALP) activity is reduced in iMEF-simBC3 cells. Subconfluent iMEF-simBC3 and iMEF-siControl cells were infected with AdWnt3A, AdBMP9, AdGFP, or AdWnt3A+AdBMP9. At day 5 post infection, cells were fixed for ALP histochemical staining assay. Each assay conditions were done in triplicate. Representative results are shown. (**B**) Wnt3A and/or BMP9-induced ALP activity is decreased in the β-catenin silenced iMEFs. The experiments were set up in a similar fashion to that described in (**A**). At days 3 and 5, cells were lysed for quantitative ALP activity assays. Basal ALP activities (e.g., GFP groups) were subtracted from all BMP9, Wnt3A, and Wnt3A+BMP9 groups. Each assay conditions were done in triplicate. “**”, *p<0.001* (iMEF-simBC3 *vs*. iMEF-siControl).

We further analyzed the functional consequences of β-catenin knockdown on MSC differentiation. We and others demonstrated that canonical Wnt signaling can induce osteogenic differentiation of mesenchymal stem cells [39], [40], [48], [60]. We sought to determine if Wnt3A can induce early osteogenic marker alkaline phosphatase (ALP) activity in iMEFs, and if the induced ALP activity would be reduced when β-catenin is silenced in iMEFs. We found that Wnt3A effectively induced ALP activity in iMEF-siControl cells, which was significantly blunted in iMEF-simBC3 cells (**Figure 5A**). We previously demonstrated that BMP9 is one of the most potent osteogenic BMPs in mesenchymal cell stems [26], [30], [32], [33], [61]. We found that BMP9 stimulated robust ALP activity in iMEF-siControl cells while the BMP9-induced ALP activity was remarkably reduced in iMEF-simBC3 cells (**Figure 5A**).

We previously showed that Wnt3A and BMP9 act synergistically in regulating osteogenic differentiation of MSCs [40]. We found that the iMEF-siControl cells co-transduced with Wnt3A and BMP9 exhibited higher ALP activity than that of the cells transduced with either Wnt3A or BMP9 alone, which was remarkably blunted by β-catenin knockdown (**Figure 5A**). Quantitative ALP activity analysis revealed a similar trend, BMP9 and Wnt3A+BMP9 stimulated ALP activities were significantly inhibited by β-catenin knockdown *p<0.001* (iMEF-simBC3 *vs*. iMEF-siControl) (**Figure 5B**). Thus, these results suggest that β-catenin may play an important role in this synergistic action between BMP9 and Wnt3A in osteogenic differentiation of MSCs.

### BMP9-induced ectopic bone formation from iMEFs is potentiated by Wnt3A but attenuated by β-catenin knockdown

**Figure 6:**
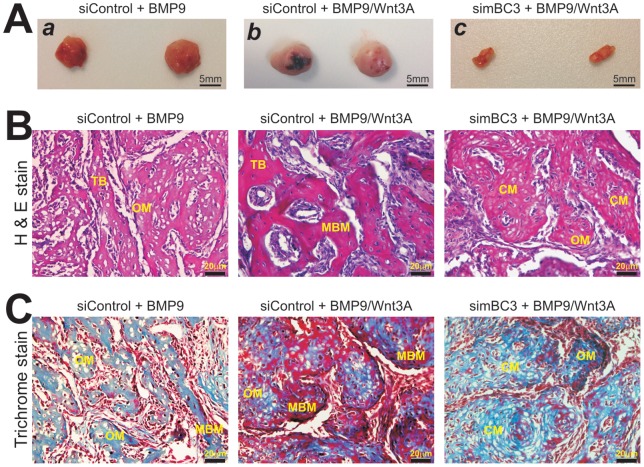
BMP9-induced ectopic bone formation from iMEFs is potentiated by Wnt3A but attenuated by β-catenin knockdown. (**A**) Gross images. Subconfluent iMEF-simBC3 and iMEF-siControl cells were infected with AdBMP9, AdWnt3A, AdBMP9+AdWnt3A, or AdGFP for 16 h. Cells were collected for subcutaneous injections (3×10^6^ cells/site in 100 μl PBS) into the flanks of athymic nude mice (n = 5 each group). At 4 weeks after injection, the animals were sacrificed. Masses formed at the injection sites were retrieved from the groups injected with the iMEF-siControl cells transduced with BMP9 (***a***) or BMP9+Wnt3A (***b***), while very small masses were retrieved from the animals injected with the iMEFs transduced BMP9+Wnt3A (c) or BMP9 (not shown). No masses were retrieved from the animals injected with Wnt3A- or GFP-transduced iMEF cells. Representative results are shown. (**B**) and (**C**) Histologic evaluation and Trichrome staining. The retrieved samples were decalcified and subjected to paraffin-embedded sectioning for histologic evaluation, including H & E staining (***B***) and Trichrome staining (***C***). Representative results are shown. TB, trabecular bone; MBM, mineralized bone matrix; OM, osteoid matrix; CM, chondroid matrix.

Using our previously established stem cell implantation assays [20], [25], [26], [30], [38], [47], [52], [57], we tested the *in vivo* effect of β-catenin knockdown on BMP9 and Wnt3A-induced ectopic bone formation. Subconfluent iMEF-simBC3 and iMEF-siControl cells were transduced with AdBMP9, AdWnt3A, AdBMP9+AdWnt3A, or AdGFP, and injected subcutaneously into the flanks of athymic nude mice for 4 weeks. No recoverable masses were detected in the GFP or Wnt3A group. Robust bony masses were retrieved from both BMP9 and BMP9+Wnt3A transduced iMEF-siControl groups, while significantly smaller masses were recovered from the iMEF-simBC3 group (**Figure 6A** **, panels a & b** ***vs.*** **c**). BMP9+Wnt3A group formed a slightly larger bone masses (**Figure 6A** **, panels a** ***vs.*** **b**).

When the retrieved samples were subjected to H & E staining, we found that BMP9-transduced iMEF-siControl cells formed evident trabecular bone, which was even more robust in the presence of both BMP9 and Wnt3A (**Figure 6B**). However, silencing β-catenin expression in iMEF-simBC3 cells significantly reduced trabecular bone formation induced by BMP9 or BMP9+Wnt3A, and formed cartilage-like small masses (**Figure 6B**). Trichrome staining confirmed that iMEF-siControl cells transduced with BMP9 formed apparently mature and mineralized bone matrices, while a combination of BMP9 and Wnt3A induced more mature and highly mineralized bone matrices (**Figure 6C**). However, the maturity and mineralization were significantly diminished in iMEF-simBC3 cells transduced with either BMP9 or BMP9+Wnt3A (**Figure 6C**). Taken together, these in vivo results strongly suggest that β-catenin may play an important role in mediating BMP9-induced bone formation, and the BMP9-Wnt3A may crosstalk in inducing osteoblastic differentiation of MSCs.

## Discussion

To overcome the technical challenges in simultaneously expressing multiple siRNAs that silence one specific gene or different genes, here we sought to develop a simple, efficient and versatile method to express multiple siRNAs in a single vector by exploring the possible utility of the Gibson DNA Assembly. We take advantages of our previously established pSOS system, in which the siRNA duplexes are generated from oligo templates driven by opposing U6 and H1 promoters [15]. Since there are only a few Pol III promoters that are well characterized, we choose to use the same U6-H1 promoter cassette to drive the expression of multiple siRNA sites. However, the use of these repetitive U6-H1 expression units poses a technical challenge for choosing overlapping sequences for Gibson DNA Assembly. To overcome this hurdle we design an assembly scheme that takes advantages of the unique sequences of different siRNA sites (e.g., stretches of 19 nucleotides). The assembly fragments containing multiple siRNA sites are generated by PCR amplifications using the back-to-back U6-H1 promoter vector pB2B as a template while the vector is SwaI-linearized pSOK.

We carried out the proof-of-principle studies using multiple siRNAs targeting human and mouse β-catenin. We demonstrate that the assembly reactions are efficient, and that candidate clones are readily identified by PCR screening. Functional analyses demonstrate that multiple β-catenin siRNA constructs can effectively silence endogenous β-catenin expression, inhibit Wnt3A-induced β-catenin/Tcf reporter activity and the expression of Wnt/β-catenin downstream target genes. Furthermore, in mesenchymal stem cells we found that silencing β-catenin inhibits Wnt3A-induced early osteogenic differentiation and significantly diminishes the synergistic osteogenic activity between BMP9 and Wnt3A both *in vitro* and *in vivo*. Therefore, our results have demonstrated that the Gibson Assembly-based pSOK system is proven simplistic, effective and versatile for simultaneous expression of multiple siRNA target sites.

Our findings have addressed at least two technical and functional concerns over the pSOK system. First, our design for the overlapping ends of the inserts is only 19 nucleotides. It's conceivable the overlapping sequences are too short and may comprise the assembly reactions. Our results indicate that the assembly efficiency is reasonably high although, in our attempt to assemble four siRNA sites, we do obtain clones that contain one, two or three sites. In fact, vectors containing three siRNAs are seemingly more favorably assembled under our assembly condition. Second, the repeated U6-H1 promoter cassettes may compromise the expression of multiple siRNA sites due to possible promoter competition [62]. Our results using the clones containing different numbers of siRNA sites strongly suggest that the use of repetitive U6-H1 expression cassettes may pose little or insignificant impact on the efficient expression of multiple siRNA sites although we did not analyze the precise expression levels of these siRNA duplexes. Given the nature of the short 19-nt overlapping sequences, we have found two critical technical parameters should be taken into consideration for an efficient assembly: 1) using 3∼5 times more inserts than conventional ligation reactions; and 2) using shorter assembly reaction time (e.g., 30–45 min at 50°C). Furthermore, it is conceivable that the same assembly system can be introduced into recombinant adenovirus, adenovirus-associated virus, and other gene delivery vector systems.

In this study, we examined the functional consequences of β-catenin knockdown on Wnt3A and/or BMP9-induced osteogenic differentiation of mesenchymal stem cells. Wnts are a family of secreted glycoproteins that regulate many developmental processes [63]. Wnt signaling plays an important role in skeletal development [60], [64]. Wnt proteins bind to their cognate receptor frizzled (Fz) and LRP-5/6 co-receptors, and activate distinct signaling pathways, including the canonical β-catenin pathway. In the absence of Wnt signaling, β-catenin is degraded by the proteasome system after GSK3β dependent phosphorylation. In the presence of Wnt signaling, unphosphorylated β-catenin accumulates in the cytoplasm and translocates into the nucleus where it associates with Tcf/LEF transcription factors to regulate the expression of target genes [59], [65]–[67]. However, the precise function of Wnt/β-catenin in osteoblastic differentiation remains to be fully elucidated. We previously found that BMP9 (aka, GDF2) is one of the most potent osteogenic BMPs [26], [30], [32], [33], [61], [68]. Through gene expression profiling, we found that Wnt3A and BMP9 regulated the expression of overlapping but distinct sets of downstream target genes in MSCs [39], [48], suggesting that there may be an important crosstalk between BMP and Wnt-induced osteogenic signaling. In this study, we used iMEFs and demonstrated that Wnt3A and BMP9 can potentiate each other's ability to induce osteogenic differentiation *in vitro* and *in vivo*. Furthermore, β-catenin knockdown significantly diminishes BMP9-induced osteogenic differentiation of iMEFs, indicating that BMP9-induced osteoblastic differentiation requires functional β-catenin signaling.

In summary, we provide a conceptual design of a simplified and versatile system for the simultaneous expression of multiple siRNAs that silence one or different genes. A series of proof-of-concept studies have validated the technical feasibility and functional efficiency of the pSOK system by silencing human and mouse β-catenin expression. Thus, our results have demonstrated that the GDA-based pSOK system should be a valuable tool for gene function studies and the development of therapeutics.

## Supporting Information

Figure S1 Schematic representations of the pSOK and pB2B vectors developed in this study. (**A**) The pSOK vector is a Murine Stem Cell Virus (MSCV) retroviral vector. It was derived from the previously developed pSOS vector [15]. The pSOK is a destination vector used for the one-step Gibson Assembly after SwaI linearization. This vector confers Blasticidin S resistance for generating stable mammalian cell lines. (**B**) The pB2B vector is a common template for PCR amplifications to generate the fragments with distinct siRNA target sites, which are subsequently used for Gibson Assembly with the SwaI-linearized pSOK vector. The full-length sequences and maps of these vectors are available at: http://www.boneandcancer.org/MOLab%20Vectors%20after%20Nov%201%202005/pSOK.pdf and http://www.boneandcancer.org/MOLab%20Vectors%20after%20Nov%201%202005/pBOK%20vector%20map%20and%20sequence%202013-12-02.pdf.
(TIF)

Figure S2 A Guide for primer design and essential sequences for assembly analysis. (**A**) Primer design guide. To make a construct containing four siRNA target sites driven by opposing U6-H1 promoters, three PCR fragments will be made for the assembly reaction. Please note the sense-strand (upper case; driven by U6 promoter) and reverse-complement strand (lower case) of the chosen siRNA sites. (**B**) The DNA sequence of the H1-U6 back-to-back promoters in pB2B is used to amplify the different siRNA fragments. Please note the template sequence contains the “TTTTT” and “AAAAA” sequences to terminate siRNA transcripts. (**C**) The assembled query sequence for BLAST analysis of sequenced candidadte clones. One can simply replace the designed “X”, “Y” and “Z” target site sequences (red and underlined) and use the modified sequence as a template to perform BLAST2 analysis and verify colony authenticity.
(TIF)

Figure S3 Function validation of the silencing efficiency of four siRNA sites targeting human β-catenin. 293 and SW480 cells stably expressing one, two, three, four siRNA sites, or siControl were generated as described in Methods. Subconfluent 293 lines were co-transfected with TOP-Luc and pCMV-Wnt3A plasmids (**A**) while the SW480 lines were just transfected with TOP-Luc reporter plasmid (**B**). At 24 h and 48 h after transfection, cells were lysed and subjected to firefly luciferase activity assays as described in Methods. Each assay condition was done in triplicate.
(TIF)

Table S1 Primers used for PCR analysis.
(XLS)
