# Supplementary figures and images for: A Simplified and Versatile System for the Simultaneous Expression of Multiple siRNAs in Mammalian Cells Using Gibson DNA Assembly
Source: PLoS One. 2014 Nov 14;9(11):e113064. doi: 10.1371/journal.pone.0113064 (PMC4232585; doi:10.1371/journal.pone.0113064)

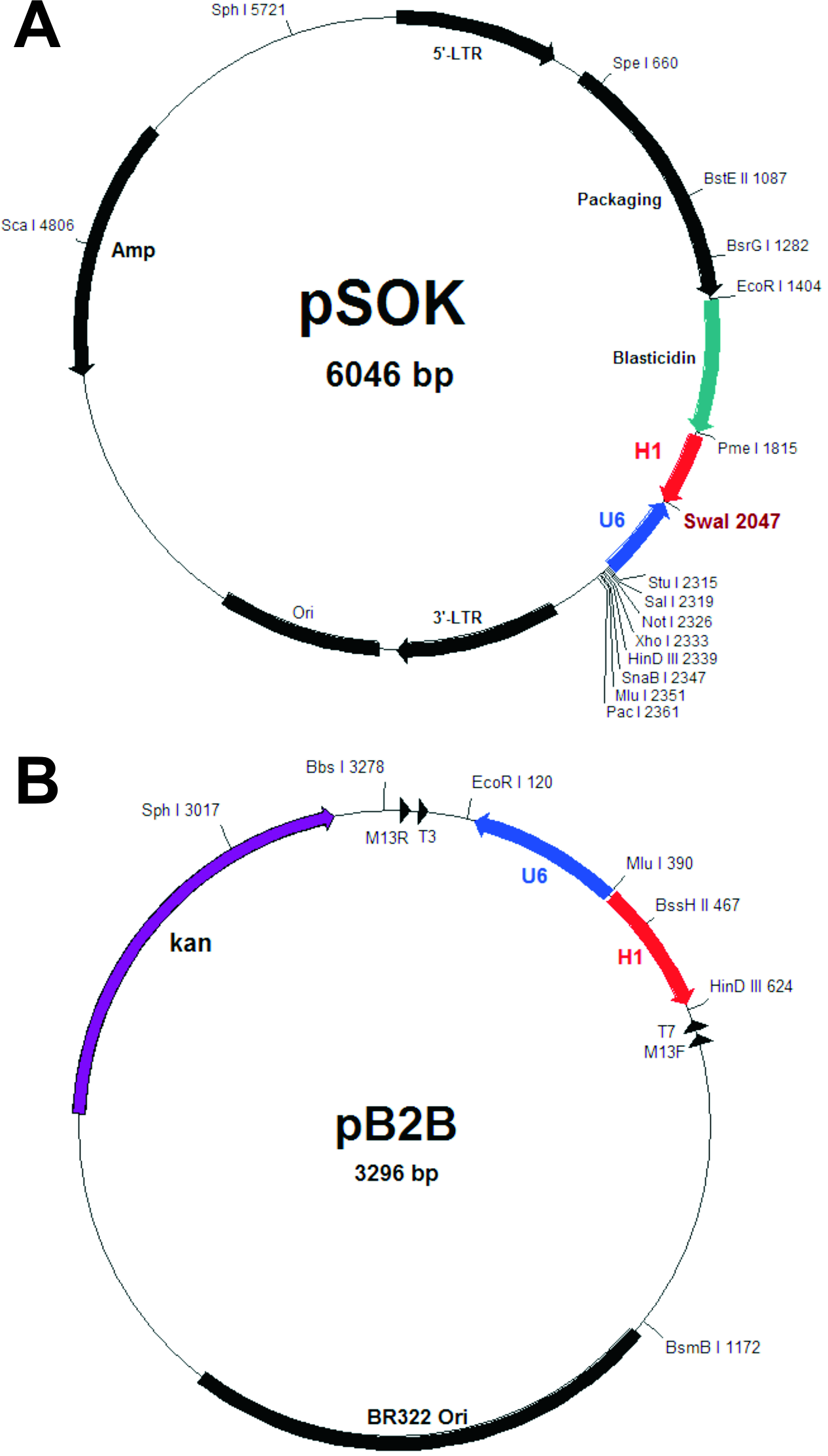

Supplement: Figure S1 — Schematic representations of the pSOK and pB2B vectors developed in this study. (A) The pSOK vector is a Murine Stem Cell Virus (MSCV) retroviral vector. It was derived from the previously developed pSOS vector [15]. The pSOK is a destination vector used for the one-step Gibson Assembly after SwaI linearization. This vector confers Blasticidin S resistance for generating stable mammalian cell lines. (B) The pB2B vector is a common template for PCR amplifications to generate the fragments with distinct siRNA target sites, which are subsequently used for Gibson Assembly with the SwaI-linearized pSOK vector. The full-length sequences and maps of these vectors are available at: http://www.boneandcancer.org/MOLab%20Vectors%20after%20Nov%201%202005/pSOK.pdf and http://www.boneandcancer.org/MOLab%20Vectors%20after%20Nov%201%202005/pBOK%20vector%20map%20and%20sequence%202013-12-02.pdf. (TIF) [file pone.0113064.s001.tif]

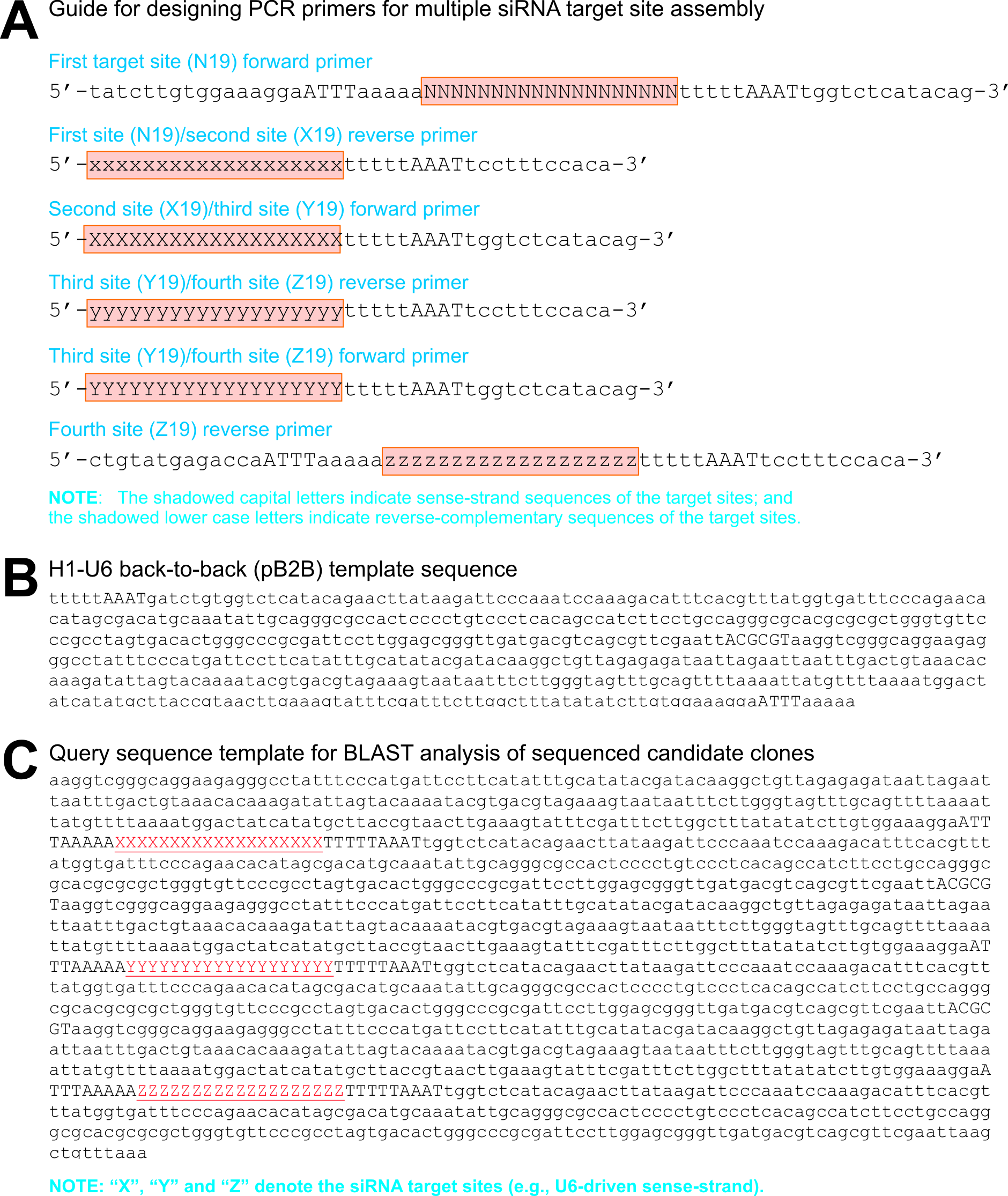

Supplement: Figure S2 — A Guide for primer design and essential sequences for assembly analysis. (A) Primer design guide. To make a construct containing four siRNA target sites driven by opposing U6-H1 promoters, three PCR fragments will be made for the assembly reaction. Please note the sense-strand (upper case; driven by U6 promoter) and reverse-complement strand (lower case) of the chosen siRNA sites. (B) The DNA sequence of the H1-U6 back-to-back promoters in pB2B is used to amplify the different siRNA fragments. Please note the template sequence contains the “TTTTT” and “AAAAA” sequences to terminate siRNA transcripts. (C) The assembled query sequence for BLAST analysis of sequenced candidadte clones. One can simply replace the designed “X”, “Y” and “Z” target site sequences (red and underlined) and use the modified sequence as a template to perform BLAST2 analysis and verify colony authenticity. (TIF) [file pone.0113064.s002.tif]

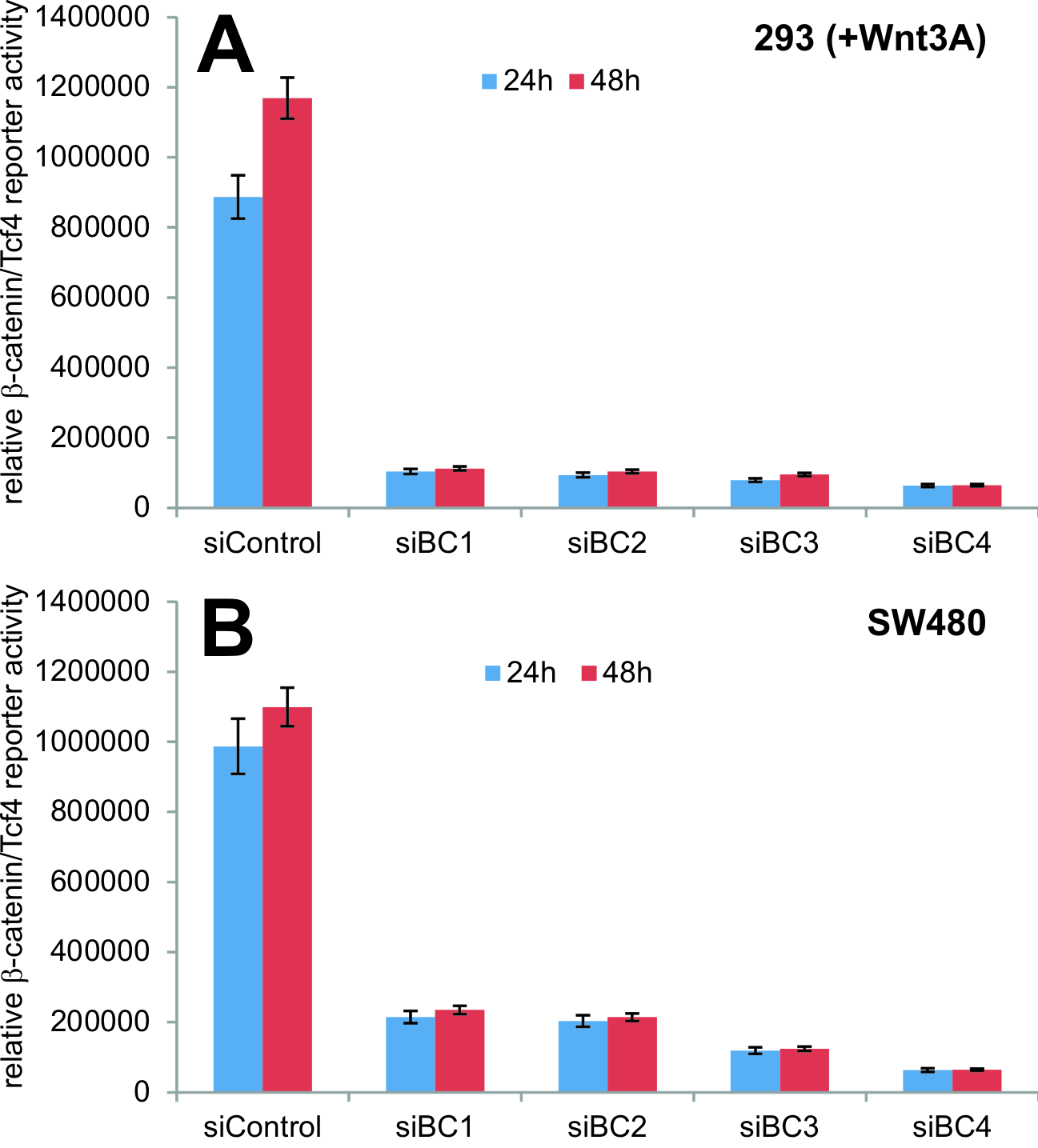

Supplement: Figure S3 — Function validation of the silencing efficiency of four siRNA sites targeting human β-catenin. 293 and SW480 cells stably expressing one, two, three, four siRNA sites, or siControl were generated as described in Methods. Subconfluent 293 lines were co-transfected with TOP-Luc and pCMV-Wnt3A plasmids (A) while the SW480 lines were just transfected with TOP-Luc reporter plasmid (B). At 24 h and 48 h after transfection, cells were lysed and subjected to firefly luciferase activity assays as described in Methods. Each assay condition was done in triplicate. (TIF) [file pone.0113064.s003.tif]
